# Public opinion survey on heritable human genome editing in South Africa: a study protocol

**DOI:** 10.1080/16549716.2025.2598132

**Published:** 2026-01-02

**Authors:** Siddharthiya Pillay, Donrich Thaldar

**Affiliations:** School of Law, University of Kwazulu-Natal, Durban, South Africa

**Keywords:** Heritable genome editing, public opinion, bioethics, policy preferences, pandemic

## Abstract

Heritable human genome editing (HHGE) presents new possibilities for the prevention of genetic diseases but also raises ethical and societal questions. While international surveys have explored public attitudes, particularly in high-income countries, there is a lack of large-scale empirical data from the Global South. In South Africa, previous work used deliberative public engagement to examine public perspectives. The present study aims to complement this by capturing public opinion through a cross-sectional survey, enabling direct comparison with deliberative findings. This study will recruit 400 adult participants residing in South Africa using targeted Facebook advertisements. A two-phase sampling process will be employed: initial screening for demographic information, followed by stratified sampling to ensure a representative South African population. The opinion survey consists of 19 HHGE scenarios, each explored through private and public moral lenses. Additionally, participants will indicate their interpretation of ‘safe and effective’ genome editing. Quantitative data will be analysed using descriptive statistics, chi-square tests, and logistic regression. Qualitative responses will undergo thematic analysis using both manual coding and generative AI tools under human oversight. The study includes two stages of informed consent and ensures data confidentiality through strict data handling protocols. Results will be disseminated in peer-reviewed journals and policy forums. The study will also generate a secondary dataset for evaluating AI-assisted qualitative analysis, to be conducted under separate ethical clearance.

## PAPER CONTEXT

- **Added knowledge**: As a study design article, its methodological knowledge may guide future research designs on virtual public opinion surveys for heritable human genome editing.
- **Global health impact for policy and action**: As a study design article, this does not directly contribute to policy and action except to add methodological knowledge on how to ascertain public opinion via online survey for HHGE. However, the actual study will provide empirical knowledge on public opinion on HHGE applications for health-related matters, which may guide policymakers.

## Background

Heritable human genome editing (HHGE) is a ground-breaking biotechnology that enables the modification of human DNA at the embryonic stage, with changes passed down to future generations. This technology holds the potential to prevent serious genetic disorders and reduce disease burdens, but it also raises urgent ethical, legal, and societal concerns [1]. Public opinion can affect the adoption of gene editing technologies [2]. As countries move toward establishing governance frameworks for HHGE, understanding public attitudes has become a critical component of responsible policy development [2].

Internationally, studies on public opinion elucidate the contestations around HHGE application. Participants were concerned about who HHGE will affect and for what it will be used [1]. As such, these opinions varied based on morality, risk perception, and benefit perception [2,3], as well as whether HHGE would be used specifically to prevent severe genetic diseases, protect against infectious disease, or used for enhancements [3,4]. These surveys revealed broad support for therapeutic applications of HHGE, particularly those aimed at preventing serious genetic diseases [3,4] In contrast, there is significantly less support for non-therapeutic enhancements, such as modifications related to intelligence or appearance [3–5]. These trends suggest that the public tends to support HHGE when framed as a tool for alleviating suffering but resists applications seen as enhancing traits beyond species-typical norms.

While these findings are informative, they largely reflect the views of populations in high-income countries, such as the Netherlands [1,4], Canada, the US, Austria, Germany and Italy [2], Japan [3] and the United States of America [5]. There is a notable absence of large-scale public opinion data from the Global South, particularly from African countries, where the socio-economic context and health priorities may shape public views differently [6]. In low- to middle-income countries, for example, support for genome editing to enhance immunity to infectious diseases has been higher, aligning with local disease burdens [6]. Jibrilla et al. [7] provide one pertinent example of a study on public opinion in Nigeria, although they do not focus on HHGE. Their findings show that there is generally support for gene editing, particularly to prevent or reduce the risk of severe diseases [7]. It is also important to note that none of the existing studies frame the policy scenarios in light of the recent pandemic, i.e. the COVID-19 pandemic, to establish whether the aftermath of this may affect public sentiment towards using HHGE to prevent possible future pandemics caused by emerging pathogens.

In South Africa, the earliest empirical studies of public opinion on HHGE were conducted by Thaldar et al. [8,9] through deliberative public engagement. These studies found strong public support for the use of HHGE to prevent severe heritable diseases and enhance immunity to major infectious diseases, such as tuberculosis (TB) and HIV/AIDS, which may correlate to the disease burden observed in South Africa. However, support declined markedly for editing aimed at less serious conditions, disabilities, or non-medical traits such as eye colour or intelligence. Importantly, these findings were based on small groups that were informed about HHGE, as per Thaldar et al.’s [8] protocol, and deliberated their views on a facilitated platform. Thus, these views may not reflect broader public sentiment where people are not intentionally informed about HHGE nor have the opportunity to deliberate with individuals who have different perspectives.

The current study addresses this gap by conducting a large-scale, cross-sectional survey of public opinion on HHGE among South Africans. By using the same 15 scenarios from the Thaldar et al. deliberative engagement study – plus five additional or amended scenarios exploring perceptions linked to pandemic preparedness and to atypical species’ characteristics – this survey enables direct comparison between informed and uninformed views. In doing so, it contributes both nationally and globally to the understanding of public attitudes toward HHGE applications, while also highlighting how local socio-cultural and health factors shape perspectives on emerging biotechnologies.

## Research objectives

The study is guided by the following research objectives:

1. To determine the level of public support for potential HHGE applications in the future aimed at specific health scenarios, including:
  - Editing to correct genetic errors to prevent categories of genetic conditions, ranging in the severity of their health-related symptoms or complications and/or sociocultural implications in the South African setting;
  - Editing to confer immunity against categories of infectious diseases which range in the severity of their health-related symptoms or complications, with a sub-focus on editing to prepare a population for hypothetical pandemic scenarios;
  - Editing to influence non-health-related traits, also referred to as enhancement.
2. To examine demographic variations in public opinion, by analysing responses according to population group, age, sex, and education level.
3. To compare:
  - Spontaneous public opinion captured in this study with deliberative public views reported by Thaldar et al. [9].
  - Private moral choices (what individuals would choose for their own child) with public moral views (what individuals think the law should allow for society at large).
4. To assess public perspectives on the temporal focus of science policy, specifically whether it should focus solely on what is currently safe and effective or also consider future scientific developments that may become safe and effective.
5. To identify public interpretations of what constitutes ‘safe and effective’ HHGE and examine how these interpretations influence participants’ support for various applications of genome editing.
6. To contribute to the global literature on HHGE, by:
  - Identifying alignments or divergences between South African public opinion and trends observed in high-income countries.
  - Exploring the influence of local health challenges and socio-cultural contexts on attitudes toward HHGE.

By achieving these objectives, the study will provide robust empirical evidence to inform policy, ethical debates, and governance frameworks on genome editing in South Africa and comparable settings.

## Methods and analysis

This section outlines the methodological framework for the study, detailing the design, sampling, data collection instruments, and analysis strategies. The study uses a mixed-methods approach to collect both quantitative and qualitative data on public opinion regarding HHGE in South Africa. By integrating structured survey responses with open-ended qualitative input, the study aims to capture both measurable trends and nuanced perspectives. All procedures are designed to ensure ethical integrity, data quality, and demographic representativeness. This study has been granted institutional ethical clearance and will adhere to the principles of the Declaration of Helsinki.

### Study design

This study employs a cross-sectional survey design to measure spontaneous public opinion on various potential applications of HHGE among adult residents of South Africa. The survey is conducted online using SurveyMonkey and is structured around 19 scenarios derived from those used in the deliberative public engagement study by Thaldar et al. [9]. Of these scenarios, 15 correspond to Thaldar et al.’s [9] previous study, and there are five new scenarios that have been added or amended. Four of these scenarios relate to pandemic preparedness, a relevant topic given the recent COVID-19 pandemic. Furthermore, an additional new scenario, involving the creation of novel eye colours, has been included to explore public perceptions of genetic modifications beyond species-typical traits. These are explained further under the sub-section for ‘Survey instrument’. The inclusion of questions on both private morality (personal choice) and public morality (policy preference) provides a dual-lens analysis of ethical reasoning. Furthermore, participants will be asked to interpret the phrase ‘safe and effective’ to examine how their conceptual understanding shapes their views on the permissibility of HHGE applications.

### Participant recruitment and data collection processes

Participants will be recruited using targeted Facebook advertisements aimed at South African residents aged 18 years and older. Facebook was selected as the primary recruitment platform due to its extensive reach across demographic groups in South Africa. As of January 2024, approximately 74.7% of South Africans aged 16 to 65 reported using the internet, and of these individuals, 88.4% reported using Facebook at least once per month [10]. Therefore, 66% of the population within this age group likely use Facebook. This makes Facebook the social media platform with the highest advertisement reach in the country, rendering it both practical and cost-effective for large-scale recruitment.

Recruitment will occur in two phases. In the first phase, individuals will be directed via Facebook ads to the study’s dedicated Facebook page, where they can access a link to the screening questionnaire hosted on SurveyMonkey. Upon accessing the survey platform, potential participants will be presented with an informed consent form detailing the purpose of the study, data use, the voluntary nature of participation, and compensation procedures. Only those who consent on this online form via writing/ticking will proceed to complete the screening questions, collecting their demographic data, including age, sex, population group, educational attainment, and religious affiliation, along with an email address. These data will be used for stratified selection to ensure the final sample is demographically representative of the South African adult population.

In the second phase, 400 participants will be selected through stratified random sampling from the pool of respondents who completed the screening questionnaire. A stratified sampling approach will ensure that the final cohort of 400 participants reflects the demographic composition of the South African adult population. This design allows for comparison across population groups, age brackets, education levels, and other demographic variables. Selected individuals will be invited to participate in the full opinion survey via personalised email, containing a unique survey link. This approach prevents multiple completions and preserves the integrity of the dataset. Participants will be given two weeks to complete the survey. Reminder emails will be sent every two days until submission. If the response threshold is not met within the timeframe, a second batch of demographically matched participants will be invited, and this process will continue until 400 completed responses are obtained.

Participants who complete the full opinion survey will be fairly compensated their mobile phone number provided at the end of the survey. Only one response per cell phone number will be accepted, and participants providing duplicate entries will be excluded based on the criteria outlined below.

### Eligibility criteria

To be eligible to participate in the study, individuals must meet the following inclusion criteria:

- Be a resident of South Africa.
- Be 18 years of age or older at the time of survey completion.
- Have access to the internet and be able to complete an online survey using a smartphone, computer, or similar device.
- Provide informed consent via the online forms to participate in the study, regardless of whether they are selected for the opinion survey phase.

For participation in the opinion survey phase specifically, selected individuals must also:

- Complete all sections of the opinion survey.
- Provide a valid South African cell phone number to facilitate compensation.

Participants will be excluded from the study if they:

- Do not meet the above inclusion criteria.
- Submit multiple responses using the same cell phone number – only the first complete response will be retained; subsequent entries from the same number will be excluded.
- Attempt to bypass the one-response rule by using different phone numbers, IP addresses, or browser profiles (measures such as IP tracking and browser restrictions will be enabled via SurveyMonkey to minimise this).
- Provide a non-South African cell phone number, indicating they are not residing in South Africa.
- Exhibit suspicious response patterns, such as unrealistically fast completion times or duplicative data entries.

These eligibility criteria are designed to ensure that the participant pool is both representative of the South African adult population and composed of genuine, engaged respondents.

### Sample size justification

The target sample size for this study is 400 participants. This figure was selected to achieve a balance between statistical robustness and practical feasibility. Given South Africa’s estimated adult population of approximately 46 million, a sample of 385 participants would typically be sufficient to ensure a 95% confidence level with a ±5% margin of error [11]. By increasing the sample size to 400, the study enhances the precision, reliability, and representativeness of its findings. This sample size also allows for meaningful subgroup analyses across key demographic variables, such as population group, age, sex, education level, and religious affiliation. Additionally, it supports the comparison between spontaneous public opinion and the informed deliberative views captured in previous studies by Thaldar et al. [9]. The sample size was chosen to accommodate potential variability in response patterns, ensure adequate statistical power for inferential tests, and reflect the diversity of South Africa’s adult population while maintaining an efficient, cost-effective research process.

### Stratified sampling and randomisation

A stratified sampling approach will be employed to achieve demographic representativeness of South Africa’s adult population. Stratification will be based on self-reported demographic characteristics collected during the screening phase, including age, sex, population group, education level, and religious affiliation (see Supplementary Materials for the full instrument). These strata reflect key sociodemographic variables used in national statistics and ensure that diverse population segments are proportionally represented in the final sample.

Within each stratum, participants will be randomly selected to reduce selection bias and maintain statistical rigour. Where minority demographic groups may otherwise be underrepresented, limited oversampling will be applied, followed by weighting adjustments during analysis to preserve overall representativeness. Replacement sampling will be conducted within strata to compensate for non-response until the target of 400 completed surveys is achieved. This stratified and randomised approach is designed to produce a sample that captures the heterogeneity of South African public opinion while balancing practical feasibility with methodological robustness.

### Pre-survey educational materials

To encourage participants to develop a basic understanding of key scientific concepts, respondents will be invited to view short, optional educational videos explaining the fundamentals of genetics and genome editing. The materials provide factual background on DNA, genes, and CRISPR-Cas9 without advancing any ethical positions that could bias participant responses. A full list of video titles and links is provided in the Supplementary Materials.

Although the educational materials were not formally tested for neutrality, they were reviewed by the multidisciplinary research team to ensure factual accuracy and non-evaluative content. Two videos provide simple, introductory explanations of genes and DNA, and the third – produced by the Mayo Clinic – describes how CRISPR works without presenting value-laden or speculative claims. These materials are included solely to provide a basic shared understanding, not to influence participant views.

The use of pre-survey educational content reflects the study’s commitment to transparency and comprehension while maintaining the non-deliberative nature of the data collection. Participants may proceed directly to the survey regardless of whether they view the materials, preserving the focus on spontaneous public attitudes that are typical of a democratic society. At the end of the questionnaire, participants will indicate whether they watched the videos, allowing for exploratory analysis of whether prior engagement with scientific information influences responses.

### Survey instrument

The survey instrument comprises two components: a demographic screening questionnaire and the main opinion survey assessing public views on HHGE. Optional open-text fields are included throughout to capture qualitative insights. Only respondents who complete the screening questionnaire and provide informed consent via the online form are considered for inclusion in the opinion survey. Collectively, the instrument is designed to elicit structured, comparative data while allowing for nuanced interpretation of underlying values through optional free-text responses.

The opinion survey (see Supplementary Materials) is organised into four sections:

1. **Interpretation of ‘safe and effective’** – examines how participants conceptualise safety in the context of HHGE by selecting among different definitions and, if desired, elaborating in an open-text response. To account for variability in how participants interpret ‘safe’ and ‘effective,’ the survey provides three predefined interpretive options for each term, based on widely accepted scientific and ethical understandings. Participants may also select ‘Other’ and provide their own explanation. These free-text responses will be reviewed to determine whether they align with the predefined categories or represent distinct interpretations. Patterns in ‘Other’ responses will be analysed to assess how public understandings converge or diverge from one another and from established scientific and health ethics definitions of safety and efficacy and public views on these matters [3].
2. **Policy scenarios** – presents a range of possible HHGE applications, covering therapeutic, preventive, and enhancement purposes. Each scenario includes two decision contexts: a *private* decision (for one’s own hypothetical child) and a *public* decision (policy preference for society). This structure enables comparison between individual and collective moral reasoning.
3. **General comments** – provides space for participants to express broader reflections on HHGE.
4. **Media engagement** – records whether participants viewed the optional pre-survey educational videos or other media content related to genome editing.

The scenarios included in the survey were adapted from the structure and rationale of the original deliberative public engagement protocol by Thaldar et al. [8], which categorises applications of heritable genome editing (HHGE) into: (1) prevention of heritable genetic conditions; (2) editing for immunity; and (3) enhancement traits. This structure aligns with established international HHGE survey frameworks [7,12,13], thereby supporting comparability with global attitudinal data.

The prevention scenarios span a gradient of severity and are not context-specific, although examples are provided to guide participants’ understanding. Down’s syndrome and oculocutaneous albinism were included due to their prevalence in South Africa and the social stigma that these conditions often attract [14,15]. The editing-for-immunity scenarios follow the Thaldar et al. [8] framework and incorporate conditions of particular relevance to South Africa’s disease burden. In 2022, TB was the second leading cause of death by a single infectious agent, with one of the highest incidence rates globally (more than 10 million per year) [16]. Statistics for 2024 show that South Africa 389 people per 100,000 contracted TB, whilst it caused approximately 54,000 deaths. More than half these deaths were in people co-infected with HIV [17], an infection that affects approximately 8 million South Africans [18]. The combination of high prevalence, co-infection patterns, and associated mortality creates an epidemiological context in which editing-for-immunity represents a salient and meaningful category for public opinion research.

The original COVID-19 immunity scenario from Thaldar et al. [9] was adapted into a broader pandemic-causing disease scenario, comprising four questions. Although HIV/AIDS and TB have been epidemics in the South African context [19], these four scenarios are more hypothetical and directed towards a transnational event, such as COVID-19, which now exists in the global collective memory. These scenarios can be split according to the severity of the hypothetical pandemic and to the availability of a vaccine that confers immunity. These questions have been included to explore public opinion on using HHGE to prepare for future pandemics. This approach acknowledges COVID-19 as a recent global pandemic while also allowing respondents to consider future outbreaks where vaccines may or may not be available. Given the controversy surrounding the speed of COVID-19 vaccine development [20], the scenario provides an opportunity to assess whether HHGE is perceived as a viable alternative in the context of public health emergencies.

The enhancement scenarios – covering traits such as intelligence, athleticism, aggressiveness, cooperativeness, sexual orientation, skin tone, and eye colour – mirror both the original Thaldar et al. [8] framework and widely used categories in international surveys [7,12,13]. These traits highlight ethical questions related to parental choice, fairness, and social norms. Certain traits (e.g. skin tone) also carry specific sociocultural resonance within South Africa’s historical context, supporting their inclusion as distinct scenarios. Furthermore, to the last theme of scenarios, that is, editing for enhancement, an additional new scenario, involving the creation of novel eye colours, has been included to explore public perceptions of genetic modifications beyond species-typical traits.

The survey instrument was adapted from a previously validated questionnaire developed for an earlier study on deliberative public engagement, conducted by the same principal investigator [8,9]. In that study, the instrument underwent extensive piloting to assess clarity, comprehension, and cultural appropriateness for South African participants. The current version retains the core items from the validated instrument, with only minor contextual adaptations to align with the present study’s focus.

Given this prior piloting and validation, re-piloting the instrument was deemed unnecessary and ethically inappropriate, as it would involve re-exposing participants to nearly identical sensitive material. Instead, internal review and content validation were undertaken within the research team to ensure that all modifications preserve conceptual integrity, linguistic clarity, and cultural relevance.

### Statistical analysis

This study uses both quantitative and qualitative data analyses. Quantitative data collected from the opinion survey will be analysed using both descriptive and inferential statistical methods to evaluate public attitudes toward heritable human genome editing (HHGE) in South Africa. Descriptive statistics will be used to summarise response frequencies across the 19 HHGE scenarios, offering an overview of levels of support, conditional support, uncertainty, and opposition.

These results will also be disaggregated by key demographic characteristics to provide a demographic profile of the survey sample. To explore the relationships between demographic characteristics and participants’ attitudes toward genome editing, the study will employ chi-square tests for categorical variables and logistic regression models where appropriate. These inferential methods will allow the research team to examine how variables such as education level, population group, and religious affiliation influence support for or opposition to particular genome editing applications.

A key analytical focus of the study is the comparison between private morality (personal decisions regarding one’s own child) and public morality (policy views on what should be legally permitted). The paired responses to these questions will be systematically analysed to assess the extent to which individuals differentiate between personal choice and public regulation. This analysis will also explore whether such divergences are associated with specific demographic factors or interpretations of what constitutes ‘safe and effective’ genome editing.

All statistical analyses will be conducted using GraphPad Prism and/or SPSS, based on the analytical requirements and preferences of the research team. This approach ensures both breadth and depth in interpreting the data, allowing for general trends to be identified alongside more detailed subgroup analyses.

### Qualitative analysis

In addition to quantitative responses, the survey includes several open-ended questions designed to elicit qualitative insights into public attitudes toward HHGE. These include participants’ explanations for selecting ‘yes, but subject to certain conditions’ in response to scenario questions, reflections on the meaning of ‘safe and effective,’ and any general comments provided at the end of the survey. These qualitative data will be analysed thematically to deepen the understanding of how the South African public interprets and reasons about the ethical and social dimensions of genome editing.

Thematic analysis will be conducted using a hybrid approach that combines manual coding with the support of generative artificial intelligence (GenAI) tools. Manual coding will be performed using NVivo software, with the research team developing a coding framework based on an initial review of the data. Simultaneously, GenAI tools – specifically ChatGPT, Grok, and the AI features of NVivo – will be used to conduct parallel analyses. These tools will assist in identifying recurring patterns, emergent themes, and possible interpretive gaps. All GenAI analyses will be guided by the coding framework established by the research team, and their outputs will be treated as audit contributions within an interdisciplinary collaborative model. Final decisions about the inclusion and interpretation of codes and themes will remain with the principal investigator, who will assess the coherence, relevance, and rigour of both human- and AI-generated analyses. This dual-track strategy allows for triangulation of findings, enhances the comprehensiveness of the thematic analysis, and provides a methodological foundation for the satellite study that will compare manual and GenAI-assisted coding. The qualitative analysis is thus not only instrumental in interpreting this study’s findings but also contributes to broader methodological inquiry into the use of AI in empirical bioethics research.

### Reporting and sensitivity to cultural and religious identity

While the survey collects demographic information such as religious affiliation, these data will not be used to characterise or label specific groups. Rather, they serve to contextualise how broader social and cultural factors may shape public reasoning about heritable human genome editing (HHGE). All findings will be reported in aggregate form to reflect general trends in public opinion, without attributing specific positions or values to any religious or cultural community. This approach supports the development of culturally sensitive and inclusive policy insights while avoiding biased or sensationalist interpretations that could reinforce stereotypes or stigmatise particular groups.

### Use of AI tools for thematic analysis

This study contributes to methodological innovation by formally integrating GenAI tools into the process of qualitative data analysis. While traditional manual coding remains central to the thematic interpretation of open-text responses, GenAI tools, specifically ChatGPT, Grok, and NVivo AI, will serve as parallel analytical auditors. This approach is rooted in an interdisciplinary collaborative auditing model [21], in which each GenAI tool is treated as a non-human auditor, contributing analytical suggestions for review and critical comparison by the human research team. The use of AI tools primarily enhances efficiency (speed and scalability when handling large qualitative datasets), consistency (reducing variation among individual coders), and exploratory depth.

The inclusion of multiple GenAI applications acknowledges the variability in how different systems process qualitative data. By comparing outputs across tools, the study enables triangulation and provides a richer understanding of the data than would be possible through manual coding alone. Importantly, this strategy is not intended to replace human judgment but to augment it, thereby supporting consistency, efficiency, and breadth in theme identification, particularly across a large sample of participants. AI tools will be used only as supportive aids in the qualitative analysis, with all outputs critically assessed by the human research team.

To ensure ethical compliance and protect participant confidentiality, all AI-assisted analyses will be conducted on a de-identified dataset. Identifiable information, including names, contact details, or any traceable responses, will be removed prior to processing. Furthermore, privacy settings on the GenAI tools will be configured to prevent data retention or sharing beyond the research team.

To mitigate algorithmic bias and ensure cultural sensitivity, human coders will generate their own codes and use AI only for triangulation. AI-generated interpretations will be reviewed for cultural and linguistic appropriateness, and any problematic outputs will be documented and corrected as part of the audit trail. Discrepancies will be resolved through team deliberation, drawing on the team’s diversity in age, sex and cultural background, and refined analyses may undergo additional peer review to introduce further diverse perspectives. Final coding and interpretation decisions will rest exclusively with the human researchers.

The methodological contribution of this study lies not only in the novel application of AI in qualitative bioethics research but also in its creation of a secondary dataset for a future satellite study. That follow-on project will critically evaluate the epistemological implications, efficiency, and consistency of GenAI-assisted coding in comparison to manual coding. It will be conducted under a separate research protocol and ethical clearance.

## Data management, access, and security

All data collected in this study will be handled with strict attention to confidentiality, data integrity, and secure storage. The study involves two phases of data collection – initial screening and the main opinion survey – each governed by clear procedures for informed consent and access control.

### Informed consent and participation

Participants are required to give written informed consent via online forms at both phases of the study. The first consent form is presented prior to completing the screening questionnaire and outlines the purpose of the research, the voluntary nature of participation, and how personal and survey data will be used. Only those who provide consent may proceed with demographic screening. Participants selected for the opinion survey will be presented with a second online informed consent form before starting the main questionnaire which they will have to complete by writing or ticking the appropriate box. Participants will be informed through the informed consent process that their cell phone numbers will be used solely for issuing compensation and will be permanently deleted once compensation is completed. They will also be informed that their survey responses will not be linked to their email addresses or cell phone numbers and will be informed about how it and where it will be stored securely for research purposes. Participants will have the right to withdraw from the survey at any point before submitting their responses. Participants are informed that compensation will only be issued upon full completion of the opinion survey, and no incentives are offered for the screening phase.

### Fraud prevention and data validation

To ensure the integrity of the dataset, multiple measures will be implemented to prevent fraudulent participation, including responses from non-South Africans and bots:

- South African Residency Verification: Participants must provide a valid South African cell phone number, which will be used to distribute compensation.
- Single Submission Restriction: SurveyMonkey will be configured to allow only one response per unique cell phone number, preventing multiple entries by the same individual.
- Bot Detection Measures: SurveyMonkey’s CAPTCHA verification and automated spam detection systems will be enabled to prevent bots from submitting responses.

A limitation of the study is that restricting one submission per unique cell phone number cannot fully eliminate the possibility of duplicate participation using multiple devices. However, collecting national identification numbers or similar metrics would not be ethically or legally permissible under South Africa’s Protection of Personal Information Act (POPIA) and could not be independently verified. To mitigate this risk, the survey platform records metadata such as time stamps and response patterns to identify anomalies, and the modest incentive value further reduces motivation for fraudulent participation. While some residual risk remains, these safeguards are considered proportionate and appropriate to the study’s minimal-risk, online design.

### Storage, security and access of data

The email addresses, cell phone numbers, unique participant ID and survey responses will be stored locally on the Principal Investigator and the research assistant’s password-protected notebooks. The data stored locally will be deleted upon completion of the study and publication of the results. Only the finalised datasets, outlined below, will be stored on the university’s Figshare cloud (Yabelana), as per UKZN’s Research Data Management Policy. The data stored on the university’s cloud will be retained for a minimum of 10 years, as per policy, as deleted thereafter.

Access to research data will be carefully managed to protect participant confidentiality. Only the PI and research assistant will have access to the raw data containing the personal details (email addresses and cell phone numbers). Before analysis, identifiable data, such as email addresses or cell phone numbers, will be removed. The dataset, containing only survey responses and demographic information, will be shared securely with the statistician. This will be referred to as the ‘Analysis Dataset’ which will be for internal use, as the qualitative responses might contain indirect identifiers. Only the PI, research assistant, and statistician will have access to the Analysis Dataset, which will be deleted upon completion of the study. Thus, before publication, the research assistant will meticulously check all the qualitative responses for indirect identifiers and redact these, if any are found. The PI will provide a second round of checking. This dataset will be the ‘Publication Dataset’. This approach ensures that personal details (email addresses and cell phone numbers) are handled only for essential administrative purposes, and that all analytical and publication work is conducted using data that is not linked to these details. Only the ‘Publication Dataset’, free of personal identifiers or links to identifying information, will be uploaded to Yabelana Figshare to be retained for a minimum of 10 years, after which it will be deleted. The handling of the data in this way ensures that participants’ responses cannot be linked back to them and may give them greater confidence to participate in the study.

In terms of the qualitative responses specifically, all such data will undergo a rigorous anonymisation process prior to analysis. In addition to the two-phase consent procedure and existing confidentiality safeguards, we will implement a two-stage anonymisation protocol. First, a member of the research team will review raw text to remove or replace any direct identifiers, including names, organisations, workplaces, and specific geographic references. A second team member will then cross-check the anonymised transcripts to identify and generalise any indirect identifiers, such as unique personal circumstances or rare demographic combinations.

Demographic variables will be used only in aggregate, and no participant will be identifiable through demographic clusters. Any quotations used in reporting will be paraphrased or further generalised where there is a risk of deductive disclosure. In addition, anonymised excerpts will be reviewed collectively during team coding discussions, enabling multiple perspectives to detect residual identifying information. These steps ensure that all qualitative data are fully anonymised prior to analysis and that the risk of unintended identification is minimised.

## Discussion

The prospective study based on this protocol offers a timely and necessary intervention into the emerging field of public engagement with HHGE, particularly within underrepresented contexts such as South Africa. By capturing the spontaneous, non-deliberative views of a demographically representative population sample, it responds to growing calls for more inclusive and locally grounded data on public attitudes toward genomic technologies.

For practice, the prospective study will provide empirical insights into how South Africans interpret core ethical concepts such as safety, effectiveness, and permissibility in the context of HHGE. These findings can assist clinicians, genetic counsellors, and science communicators in better understanding how laypeople perceive risk, choice, and therapeutic intention, enabling more responsive engagement and communication strategies in clinical and community settings.

For policy, the study will offer robust public opinion data that can inform governance frameworks for genome editing in South Africa. Unlike deliberative studies that rely on informed participants, this survey captures widespread public sentiment, including moral and cultural intuitions that may otherwise remain invisible in policymaking processes. The dual framing of private and public morality further enriches the data, offering insights into how individuals differentiate between personal autonomy and societal regulation. These perspectives are essential for ethical policy development, particularly in democratic societies seeking legitimacy through public consultation.

For scholarship, the study contributes to global literature by expanding the geographic and methodological scope of public opinion research on HHGE. It introduces an innovative comparative design that links deliberative and spontaneous viewpoints, and it pioneers the integration of generative AI tools in qualitative data analysis. This methodological contribution lays the groundwork for future research into the epistemological, ethical, and practical implications of AI-assisted qualitative methods in empirical bioethics.

Through its design and scope, the study supports a more inclusive, context-sensitive, and methodologically plural understanding of how emerging biotechnologies are received by the public, which is an essential step in fostering ethically sound and socially legitimate science policy.

## Limitations

While the study is designed to maximise representativeness and rigour, several limitations must be acknowledged. There are three limitations that can be identified from the outset of the study: limitations in Facebook recruitment; the cross-sectional versus longitudinal nature of the study; and the use of GenAI in analyses.

First, the online nature of recruitment may exclude individuals without reliable internet access, particularly in rural or economically disadvantaged areas. This constitutes a limitation of the sampling approach. No supplementary recruitment strategies will be implemented beyond the online platform, and this limitation is acknowledged explicitly. Facebook advertising was selected as the primary recruitment method because it offers a cost-effective way to reach a national audience within the study’s resource constraints. Complementary recruitment strategies, particularly in rural areas, are not feasible due to the financial and logistical demands of travel, translation and gatekeeper permissions. We acknowledge that this may lead to under-representation of some groups and note this as a limitation and an avenue for future research. Nevertheless, recent national data suggest that as of January 2024, 66% of the population within the age group of 16 to 65 likely use Facebook [10]. While this figure may not capture all usage patterns, it suggests that Facebook provides extensive coverage of the general adult population, including a substantial proportion of users from diverse socioeconomic backgrounds. Given that the aim of this study is to explore public attitudes toward HHGE rather than to produce nationally representative estimates, recruitment through Facebook remains a reasonable and pragmatic approach.

Second, as a cross-sectional survey, the study captures views at a single point in time and does not account for how public attitudes may evolve through exposure to new information or public debate. The absence of a deliberative component means that changes in opinion arising from education, reflection, or dialogue are not measured within this study.

Third, while the use of generative AI tools enhances the scale and consistency of qualitative analysis, these tools may produce overly broad, generic themes, or introduce bias, without human oversight. As such, the integration of AI is exploratory and will be accompanied by critical human review. All AI-assisted coding outputs will be cross-checked against a subset of data manually coded by members of the research team to ensure consistency and accuracy. Discrepancies between human and AI coding will be discussed, and final thematic categories will be confirmed through consensus among the research team. Recognising that AI models may reproduce or amplify bias present in their training data, all outputs will be reviewed critically by human coders for contextual and cultural appropriateness, especially given the sensitivity of the South African setting. AI-generated outputs will only be accepted once these are verified by a member of the research team.

Despite these limitations, the study offers a valuable and novel dataset that will inform future research, engagement efforts, and policymaking on HHGE in South Africa and comparable contexts.

## Supplementary Material

Supplementary_Material_ clean.docx

## Data Availability

The informational video links, screening questions and opinion survey can be viewed in the Supplementary Materials. No further data is available for this study design article.
