# Supplementary material for: Public opinion survey on heritable human genome editing in South Africa: a study protocol
Source: Glob Health Action. 2026 Jan 2;19(1):2598132. doi: 10.1080/16549716.2025.2598132 (PMC12777815; doi:10.1080/16549716.2025.2598132)
Supplement: Supplementary_Material_ clean.docx [file ZGHA_A_2598132_SM7922.docx]

**Supplementary Material: Video Links**

Before beginning the opinion survey, participants will be invited to view three short, optional YouTube educational videos designed to explain foundational concepts in genetics and genome editing. These optional materials aim to provide accessible background information without introducing ethical perspectives that could bias responses. These videos were chosen because they present key scientific information using clear, simple language and engaging animation. These also void making normative or value-laden claims about the ethics or desirability of genome editing. In this way, we preserve the study’s focus on uninfluenced, non-deliberative public opinion. The selected videos are:

- **What is DNA?**
  [YouTube link: <https://www.youtube.com/watch?v=zwibgNGe4aY>]
- **What is a gene?**
  [YouTube link: <https://www.youtube.com/watch?v=5MQdXjRPHmQ>]
- **How does gene editing work (CRISPR-Cas9)?**
  [YouTube link: <https://www.youtube.com/watch?v=UKbrwPL3wXE>]

**Supplementary Material: Screening Questions**

Questionnaire - screening questions

Thank you for your interest in this survey on heritable genome editing. Should you be selected based on these screening questions, your responses will help us understand public perspectives on different uses of heritable genome editing before birth. Please ensure that you are a **South African resident and 18 years or older** before proceeding to complete the survey.

Please note that completing this questionnaire does not guarantee a place in the opinion survey on HHGE. You will **not** be compensated for completing the questionnaire. Only those who are selected to participate in the opinion survey will receive compensation.

This questionnaire consists of one section to collect your basic demographic details and will take about 2 minutes to complete.

# Demographic Information

In this section, you are asked 5 questions about yourself.

1. Age

Please confirm the year that you were born:

2. Sex

- Male
- Female

3. Population Group

- Black African
- Coloured
- Indian or Asian
- White
- Other

4. Highest level of education completed

- No formal education
- Some/all primary education
- Some secondary education
- Completed secondary education
- Diploma or Certificate
- Bachelor’s degree
- Postgraduate degree

5. Religious affiliation (select one or more options)

- African Traditional
- Christianity
- Hinduism
- Islam
- Judaism
- Not religious
- Other

Thank you for completing this survey. Please ensure that you provide us with your correct contact details below so that we may invite you to the opinion survey, should you be selected. Please note, once again, that you will not be compensated for completing this questionnaire.

Should you be interested in the results of this survey, please visit [**www.healthlaw.ukzn.ac.za**](http://www.healthlaw.ukzn.ac.za). Should you wish to withdraw your responses or if you have any questions, please contact [**ThaldarD@ukzn.ac.za**](mailto:ThaldarD@ukzn.ac.za)**.**

**Please provide your email address below should we need to contact you to participate in the opinion survey:**

*(email address)*

**Supplementary Material: Opinion Survey**

Questionnaire – Opinion survey

Thank you for taking part in this survey on heritable genome editing. Your responses will help us understand public perspectives on different uses of heritable genome editing before birth.

Please note that you will only be compensated for completing this survey in its entirety. Incomplete surveys will not be considered as completed and will not receive the stated compensation of a R250 Pick n Pay voucher.

This questionnaire consists of four sections and will take about 15 minutes to complete:

1. Question on safety and effectiveness – Your personal views on the standards that should guide HHGE
2. Scenarios – Your personal and policy views on various applications of heritable genome editing.
3. General comment – A space for any additional thoughts. (Optional)
4. Engagement with media – Questions about whether you have engaged with educational materials on this topic.

# Section 1:

**Survey Question**
In all the questions we ask in this survey, please assume that we are at a future time when using HHGE in clinics has become **safe and effective**. But how should **safe and effective** be understood? Please choose the option that best matches your view.

**Options:**

**Must Be Nearly Perfect**

The edit is only safe and effective if it almost always works and almost never causes new health problems — whether used to prevent disease or to make non-medical changes like changing eye colour.

🡪 *Example:* Imagine scientists edit the genes of an early embryo to remove a serious inherited risk. Some examples that might be considered serious risks include a BRCA1 gene mutation that could cause breast cancer later in life. Under this standard, the editing would only be seen as safe and effective if it works correctly almost every time and almost never causes unexpected health problems.

*This is the highest and most demanding standard.*

**Better Than Other Options**

The edit is safe and effective if it works better and has fewer health risks than the other options available today, such as medicines, regular screening, or preventive surgeries. For non-medical changes like eye colour, it should always carry very low health risks and reliably achieve the change.

🡪 *Example:* Today, a person who inherits a BRCA1 mutation can reduce their risk of breast cancer by having regular medical check-ups or even choosing preventive surgery. These options are not perfect — they still carry health risks and emotional costs. Under this standard, if editing an early embryo’s genes could more safely and reliably prevent breast cancer compared to these current options, the edit would be considered safe and effective.

*This standard is less demanding than the one above.*

**Lower Risk Than the Problem**

The edit is safe and effective if the risks caused by the editing are smaller than the risks caused by the disease or condition it is trying to prevent. For non-medical changes like eye colour, it should always carry very low health risks and reliably achieve the change.

🡪 *Example*: Breast cancer is a serious and sometimes life-threatening disease. If editing an embryo’s genes to remove a BRCA1 mutation carries some risks — like a chance of developing other health problems — but these risks are smaller than the serious risks of breast cancer, then the editing would be considered safe and effective under this standard.

*This is less demanding than both of the standards above.*

**Other—please explain
[Text entry]**

[Next button is only activated if a choice is made above].

# Section 2: Scenarios

**Instructions**

In this section of the questionnaire, you will be asked about 19 possible uses of heritable genome editing.* Each question consists of two parts: a private decision for your own hypothetical child, and a public policy decision for the general population.

For each case, you will answer **two questions**:

1. **Private Decision:** Would you personally choose to use heritable genome editing for your own child in this scenario?

2. **Public Policy:** Should the law allow parents in general to use heritable genome editing for their children in this scenario?

This will help us understand the difference between personal views and broader policy preferences.

* Heritable genome editing is a way of changing the genes of a baby before it is born in a way that these changes can be passed on to future generations. This is done by editing the DNA of sperm, eggs, or early embryos using advanced scientific tools like CRISPR-Cas9.

If you have not yet, we recommend that you watch these three videos on YouTube to provide you with information on DNA, genes, and heritable genome editing:

- This video explains DNA: <https://www.youtube.com/watch?v=zwibgNGe4aY>
- This video explains genes: <https://www.youtube.com/watch?v=5MQdXjRPHmQ>
- This video explains gene editing: <https://www.youtube.com/watch?v=UKbrwPL3wXE>

Click next to start with the first scenario.

[Next button is always active].

*[Note: These 19 scenarios will be presented in random order as to avoid the impression of scaling from non-trivial to trivial.]*

## Scenario 1: Serious heritable diseases

Serious heritable diseases like sickle cell anaemia and muscular dystrophy are passed down through families and are caused by genes that don’t work properly. These conditions can have severe effects on health and quality of life.

**Private Decision:**

If heritable genome editing were proven safe and effective, would you use it before **your child**’s birth to prevent your child from being born with a serious heritable disease?

- Yes
- Yes, but subject to certain conditions: [Textbox]
- Unsure
- No

**Public Policy:**

If heritable genome editing were proven safe and effective, should our country’s laws **allow parents generally** to use it before a child’s birth to prevent the child from being born with a serious heritable disease?

- Yes
- Yes, but subject to certain conditions: [Textbox]
- Unsure
- No

[Next button is only activated if a choice is made in both questions above].

--

## Scenario 2: Heritable diseases

Heritable diseases like asthma and eczema can run in families, with genes increasing the risk, but environmental factors and lifestyle also play an important role.

**Private Decision:**

If heritable genome editing were proven safe and effective, would you use heritable genome editing before **your child**’s birth to prevent your child from being born with a disease like asthma or eczema?

- Yes
- Yes, but subject to certain conditions: [Textbox]
- Unsure
- No

**Public Policy:**

If heritable genome editing were proven safe and effective, should our country’s laws **allow parents generally** to use it before their child’s birth to prevent the child from being born with a disease like asthma or eczema?

- Yes
- Yes, but subject to certain conditions: [Textbox]
- Unsure
- No

[Next button is only activated if a choice is made in both questions above].

--

## Scenario 3: Disability

Some disabilities, like deafness or blindness, can run in families and be inherited.

**Private Decision:**

If heritable genome editing were proven safe and effective, would you use it before **your child**’s birth to prevent your child from being born with a disability?

- Yes
- Yes, but subject to certain conditions: [Textbox]
- Unsure
- No

**Public Policy:**

If heritable genome editing were proven safe and effective, should our country’s laws **allow parents generally** to use it before their child’s birth to prevent the child from being born with a disability?

- Yes
- Yes, but subject to certain conditions: [Textbox]
- Unsure
- No

[Next button is only activated if a choice is made in both questions above].

--

## Scenario 4: Down’s syndrome

Down’s syndrome is a genetic condition that usually happens by chance at the time of conception.

**Private Decision:**

If heritable genome editing were proven safe and effective, would you use it before **your child**’s birth to prevent your child from being born with Down’s syndrome?

- Yes
- Yes, but subject to certain conditions: [Textbox]
- Unsure
- No

**Public Policy:**

If heritable genome editing were proven safe and effective, should our country’s laws **allow parents generally** to use it before their child’s birth to prevent the child from being born with Down’s syndrome?

- Yes
- Yes, but subject to certain conditions: [Textbox]
- Unsure
- No

[Next button is only activated if a choice is made in both questions above].

--

## Scenario 5: Albinism

Albinism is an inherited condition and affects the production of melanin, the pigment that gives colour to the skin, hair, and eyes. People with albinism have little or no pigment and may also have vision problems.

**Private Decision:**

If heritable genome editing were proven safe and effective, would you use it before **your child**’s birth to prevent your child from being born with albinism?

- Yes
- Yes, but subject to certain conditions: [Textbox]
- Unsure
- No

**Public Policy:**

If heritable genome editing were proven safe and effective, should our country’s laws **allow parents generally** to use it before their child’s birth to prevent the child from being born with albinism?

- Yes
- Yes, but subject to certain conditions: [Textbox]
- Unsure
- No

[Next button is only activated if a choice is made in both questions above].

--

## Scenario 6: Immunity against serious disease

Serious diseases like tuberculosis (TB), HIV, or AIDS are caused by infections. These diseases can be life-threatening, but with the right treatment, many people can live long and healthy lives.

**Private Decision:**

If heritable genome editing were proven safe and effective, would you use it before **your child**’s birth to make the child immune to contracting a serious disease like TB or HIV or AIDS during his/her life?

- Yes
- Yes, but subject to certain conditions: [Textbox]
- Unsure
- No

**Public Policy:**

If heritable genome editing were proven safe and effective, should our country’s laws **allow parents generally** to use it before their child’s birth to make the child immune to contracting a serious disease like TB or HIV or AIDS during his/her life?

- Yes
- Yes, but subject to certain conditions: [Textbox]
- Unsure
- No

[Next button is only activated if a choice is made in both questions above].

--

## Scenario 7: Immunity against disease

Illnesses like the flu or the common cold are usually mild and caused by viruses that spread from person to person. Most people recover quickly, but some may get sicker than others depending on their age, health, or immune system.

**Private Decision:**

If heritable genome editing were proven safe and effective, would you use it before **your child**’s birth to make your child immune to contracting an illness like the flu or a common cold during his/her life?

- Yes
- Yes, but subject to certain conditions: [Textbox]
- Unsure
- No

**Public Policy:**

If heritable genome editing were proven safe and effective, should our country’s laws **allow parents generally** to use it before their child’s birth to make the child immune to contracting an illness like the flu or a common cold during his/her life?

- Yes
- Yes, but subject to certain conditions: [Textbox]
- Unsure
- No

[Next button is only activated if a choice is made in both questions above].

--

## Scenario 8: Pandemic (deadly) with no vaccine

Imagine a new worldwide **pandemic** breaks out—an extremely contagious disease that kills one in every 50 people it infects. **No vaccine** exists for this disease.

**Private Decision:**

If heritable genome editing were proven to be safe and effective, would you choose to use it before your child’s birth to make **your child** immune to this new disease?

- Yes
- Yes, but subject to certain conditions: [Textbox]
- Unsure
- No

**Public Policy:**

If heritable genome editing were proven to be safe and effective, should the law in our country **allow parents generally** to use it before their child’s birth to make the child immune to this new disease?

- Yes
- Yes, but subject to certain conditions: [Textbox]
- Unsure
- No

[Next button is only activated if a choice is made in both questions above].

--

## Scenario 9: Pandemic (deadly) with vaccine

Imagine a new worldwide **pandemic** breaks out—an extremely contagious disease that kills one in every 50 people it infects. A **vaccine is quickly developed** that effectively provides immunity.

**Private Decision:**

If heritable genome editing were proven to be safe and effective, would you choose to use it before your child’s birth to make **your child** immune to this new disease?

- Yes
- Yes, but subject to certain conditions: [Textbox]
- Unsure
- No

**Public Policy:**

If heritable genome editing were proven to be safe and effective, should the law in our country **allow parents generally** to use it before their child’s birth to make the child immune to this new disease?

- Yes
- Yes, but subject to certain conditions: [Textbox]
- Unsure
- No

[Next button is only activated if a choice is made in both questions above].

--

## Scenario 10: Pandemic (long-lasting symptoms) no vaccine

Imagine a new worldwide **pandemic** breaks out—an extremely contagious disease. Although the risk of death is low (1 in 20 000), the disease can cause serious, long-lasting symptoms and complications in many who recover. **No vaccine** exists for this disease.

**Private Decision:**

If heritable genome editing were proven to be safe and effective, would you choose to use it before your child’s birth to make **your child** immune to this new disease?

- Yes
- Yes, but subject to certain conditions: [Textbox]
- Unsure
- No

**Public Policy:**

If heritable genome editing were proven to be safe and effective, should the law in our country **allow parents generally** to use it before their child’s birth to make the child immune to this new disease?

- Yes
- Yes, but subject to certain conditions: [Textbox]
- Unsure
- No

[Next button is only activated if a choice is made in both questions above].

--

## Scenario 11: Pandemic (long-lasting symptoms) with vaccine

Imagine a new worldwide **pandemic** breaks out—an extremely contagious disease. Although the risk of death is low (1 in 20 000), the disease can cause serious, long-lasting symptoms and complications in many who recover. A **vaccine is quickly developed** that effectively provides immunity.

**Private Decision:**

If heritable genome editing were proven to be safe and effective, would you choose to use it before your child’s birth to make **your child** immune to this new disease?

- Yes
- Yes, but subject to certain conditions: [Textbox]
- Unsure
- No

**Public Policy:**

If heritable genome editing were proven to be safe and effective, should the law in our country **allow parents generally** to use it before their child’s birth to make the child immune to this new disease?

- Yes
- Yes, but subject to certain conditions: [Textbox]
- Unsure
- No

[Next button is only activated if a choice is made in both questions above].

--

## Scenario 12: Intelligence

A person’s **intelligence** is influenced by their genes, but factors like upbringing, education, and life experiences also have a strong effect.

**Private Decision:**

If heritable genome editing were proven safe and effective, would you use it before **your child**’s birth to influence how intelligent your child will be?

- Yes
- Yes, but subject to certain conditions: [Textbox]
- Unsure
- No

**Public Policy:**

If heritable genome editing were proven safe and effective, should our country’s laws **allow parents generally** to use it before their child’s birth to influence how intelligent the child will be?

- Yes
- Yes, but subject to certain conditions: [Textbox]
- Unsure
- No

[Next button is only activated if a choice is made in both questions above].

--

## Scenario 13: Athleticism

A person’s **athletic** ability is partly influenced by their genes, but factors like training, nutrition, and environment also play an important role.

**Private Decision:**

If heritable genome editing were proven safe and effective, would you use it before **your child**’s birth to influence how athletic your child will be?

- Yes
- Yes, but subject to certain conditions: [Textbox]
- Unsure
- No

**Public Policy:**

If heritable genome editing were proven safe and effective, should our country’s laws **allow parents generally** to use it before their child’s birth to influence how athletic the child will be?

- Yes
- Yes, but subject to certain conditions: [Textbox]
- Unsure
- No

[Next button is only activated if a choice is made in both questions above].

--

## Scenario 14: Aggression

A person’s tendency toward **aggression** can be influenced by their genes, but things like upbringing, environment, stress, and life experiences also play a major role.

**Private Decision:**

If heritable genome editing were proven safe and effective, would you use it before **your child**’s birth to influence how aggressive your child will be?

- Yes
- Yes, but subject to certain conditions: [Textbox]
- Unsure
- No

**Public Policy:**

If heritable genome editing were proven safe and effective, should our country’s laws **allow parents generally** to use it before their child’s birth to influence how aggressive the child will be?

- Yes
- Yes, but subject to certain conditions: [Textbox]
- Unsure
- No

[Next button is only activated if a choice is made in both questions above].

--

## Scenario 15: cooperativeness

A person’s tendency to be **cooperative** can be influenced by their genes, but experiences like parenting, culture, education, and relationships also play a big role.

**Private Decision:**

If heritable genome editing were proven safe and effective, would you use it before **your child**’s birth to influence how cooperative your child will be?

- Yes
- Yes, but subject to certain conditions: [Textbox]
- Unsure
- No

**Public Policy:**

If heritable genome editing were proven safe and effective, should our country’s laws **allow parents generally** to use it before their child’s birth to influence how cooperative the child will be?

- Yes
- Yes, but subject to certain conditions: [Textbox]
- Unsure
- No

[Next button is only activated if a choice is made in both questions above].

--

## Scenario 16**:** Sexual orientation

A person’s sexual orientation—such as being straight, bisexual, or gay—is likely shaped by a mix of factors. Genes may play a role, but so can other factors that scientists are still trying to understand.

**Private Decision:**

If heritable genome editing were proven safe and effective, would you use it before **your child**’s birth to influence the sexual orientation of your child?

- Yes
- Yes, but subject to certain conditions: [Textbox]
- Unsure
- No

**Public Policy:**

If heritable genome editing were proven safe and effective, should our country’s laws **allow parents generally** to use it before their child’s birth to influence the sexual orientation of the child?

- Yes
- Yes, but subject to certain conditions: [Textbox]
- Unsure
- No

[Next button is only activated if a choice is made in both questions above].

--

## Scenario 17: Skin tone

A person’s **skin tone**—whether lighter or darker—is mostly determined by their genes.

**Private Decision:**

If heritable genome editing were proven safe and effective, would you use it before **your child**’s birth to determine your child’s skin tone?

- Yes
- Yes, but subject to certain conditions: [Textbox]
- Unsure
- No

**Public Policy:**

If heritable genome editing were proven safe and effective, should our country’s laws **allow parents generally** to use it before their child’s birth to determine the child’s skin tone (lighter or darker)?

- Yes
- Yes, but subject to certain conditions: [Textbox]
- Unsure
- No

[Next button is only activated if a choice is made in both questions above].

--

## Scenario 18: Eye colour – existing kinds of eye colour

A person’s **eye colour**—within the typical human range, like brown, green, or blue—is determined by their genes.

**Private Decision:**

If heritable genome editing were proven safe and effective, would you use it before **your child**’s birth to select your child’s eye colour within the typical human range?

- Yes
- Yes, but subject to certain conditions: [Textbox]
- Unsure
- No

**Public Policy:**

If heritable genome editing were proven safe and effective, should our country’s laws **allow parents generally** to use it before a child’s birth to select the child’s eye colour within the typical human range?

- Yes
- Yes, but subject to certain conditions: [Textbox]
- Unsure
- No

[Next button is only activated if a choice is made in both questions above].

--

## Scenario 19: Eye colour – new kinds of eye colours

Imagine a future where heritable genome editing has advanced to the point where parents can choose eye colours for their children that go beyond the typical human range—such as black, yellow, or violet.

**Private Decision:**

If heritable genome editing were proven to be safe and effective, would you choose to use it before **your child**’s birth to give your child an eye colour of your preference that is beyond the typical human range?

- Yes
- Yes, but subject to certain conditions: [Textbox]
- Unsure
- No

**Public Policy:**

If heritable genome editing were proven to be safe and effective, should the law in our country allow **parents in general** to use it before their child’s birth to give the child an eye colour of the parents’ preference that is beyond the typical human range?

- Yes
- Yes, but subject to certain conditions: [Textbox]
- Unsure
- No

[Next button is only activated if a choice is made in both questions above].

--

# Section 3: General Comment

Do you have any additional thoughts or comments about the use of heritable genome editing before a child’s birth that you would like to share? We would appreciate your insights. (Optional)

[Textbox]

[Next button is always active].

--

# Section 4: Engagement with media

This section aims to understand whether you engaged with any educational materials on heritable genome editing.

1. **Did you watch the YouTube videos on genetics and heritable genome editing that we suggested?**

• Yes, I watched them and found them useful.

• Yes, I watched them but did not find them useful.

• No, I did not watch the videos.

2. **Have you engaged with any other media (e.g., articles, news reports, documentaries, podcasts, social media discussions) on this topic?** If yes, please provide details (Optional).

**[Textbox]**

Thank you for completing this survey. Please ensure that you provide us with your correct contact details so that we may compensate you for your time. Should you be interested in the results of this survey, please visit [www.healthlaw.ukzn.ac.za](http://www.healthlaw.ukzn.ac.za). Should you wish to withdraw your responses or if you have any questions, please contact [ThaldarD@ukzn.ac.za](mailto:ThaldarD@ukzn.ac.za)**.**

**Please provide your cell phone number below so that we may compensate you for completing this survey:**

*(phone number)*

[Next button is only activated if a choice is made in question 1 above, and a valid phone number is provided].

--
